# Supplementary material for: Virtual neural network-guided optimization of non-invasive brain stimulation in Alzheimer’s disease
Source: PLoS Comput Biol. 2024 Jan 17;20(1):e1011164. doi: 10.1371/journal.pcbi.1011164 (PMC10824453; doi:10.1371/journal.pcbi.1011164)
Supplement: S1 Table — The results of independent t-tests comparing outcome measures in virtual stimulation setups to those in the ADD condition. Positive values indicate a shift towards healthy control values (bold if significant), while negative values indicate a shift further away from healthy control values (in italics if significant). ** p < 0.001. (DOCX) [file pcbi.1011164.s001.docx]

| **S1 Table: Best performing setups versus ADD.** The results of independent t-tests comparing outcome measures in virtual stimulation setups to those in the ADD condition. Positive values indicate a shift towards healthy control values (bold if significant), while negative values indicate a shift further away from healthy control values (in italics if significant). ** p < 0.001 | | | | | | | | |
| --- | --- | --- | --- | --- | --- | --- | --- | --- |
| **Setup** | **Virtual time** | **Alpha1** | **Alpha2** | **Total power** | **Peak frequency** | **PLI** | **AEC** |  |
| PO8a-AF3c vs. ADD | t=10 | **0,013 **** | **0,014 **** | **138906,350 **** | *-0,017 *** | **0,022 **** | **0,011 **** |  |
|  | t=15 | **0,098 **** | **0,056 **** | **71469,280 **** | 0,012 | **0,096 **** | **0,021 **** |  |
|  | t=20 | **0,047 **** | **0,014 **** | **8001,240 **** | **0,080 **** | **0,015 **** | **0,007 **** |  |
| PO7a-AF4c vs. ADD | t=10 | **0,013 **** | **0,015 **** | **133496,950 **** | *-0,013 *** | 0,005 | 0,011 ** |  |
|  | t=15 | **0,093 **** | **0,053 **** | **66491,200 **** | **0,018 **** | **0,102 **** | **0,020 **** |  |
|  | t=20 | **0,046 **** | **0,014 **** | **18736,703 **** | **0,081 **** | **0,017 **** | **0,007 **** |  |
| F7a-F4c vs. ADD | t=10 | **0,020 **** | **0,023 **** | **135158,490 **** | 0,006 | **0,042 **** | **0,008 **** |  |
|  | t=15 | **0,069 **** | **0,041 **** | **50323,320 **** | **0,076 **** | **0,053 **** | **0,011 **** |  |
|  | t=20 | **0,028 **** | **0,002 **** | **6187,090 **** | **0,067 **** | **0,178 **** | **0,004 **** |  |
