# Supplementary material for: Virtual neural network-guided optimization of non-invasive brain stimulation in Alzheimer’s disease
Source: PLoS Comput Biol. 2024 Jan 17;20(1):e1011164. doi: 10.1371/journal.pcbi.1011164 (PMC10824453; doi:10.1371/journal.pcbi.1011164)
Supplement: S2 Table — The results of independent t-tests comparing setups based on their difference in outcome measures from those in the ADD condition. Positive values indicate a shift towards healthy control values (bold if significant), while negative values indicate a shift further away from healthy control values (italic if significant). * p < 0.5, ** p < 0.001. (DOCX) [file pcbi.1011164.s002.docx]

| **S2 Table: Comparison of best performing setups based on difference from the ADD condition.** The results of independent t-tests comparing setups based on their difference in outcome measures from those in the ADD condition. Positive values indicate a shift towards healthy control values (bold if significant), while negative values indicate a shift further away from healthy control values (italic if significant). * p < 0.5, ** p < 0.001 | | | | | | | |
| --- | --- | --- | --- | --- | --- | --- | --- |
| **Setup** | **Virtual time** | **Alpha1** | **Alpha2** | **Total power** | **Peak frequency** | **PLI** | **AEC** |
| PO8a-AF3c vs. PO7a-AF4c | t=10 | 0,000 | 0,000 | **5409,400 *** | -0,004 | **0,017 *** | 0,000 |
|  | t=15 | 0,004 | 0,003 | **4978,080 **** | -0,006 | -0,005 | 0,000 |
|  | t=20 | 0,001 | 0,000 | 69,060 | 0,000 | -0,002 | 0,000 |
| PO8a-AF3c vs. F7a-F4c | t=10 | *-0,006 ** | *-0,009 *** | 3747,860 | *-0,023*** | *-0,019 *** | 0,003 |
|  | t=15 | **0,028 **** | **0,015 **** | **21145,960 **** | *-0,064 *** | **0,043 **** | **0,010 **** |
|  | t=20 | **0,019 **** | **0,012 **** | **1814,150 **** | 0,014 | **0,012 **** | **0,003 **** |
