# Supplementary material for: Virtual neural network-guided optimization of non-invasive brain stimulation in Alzheimer’s disease
Source: PLoS Comput Biol. 2024 Jan 17;20(1):e1011164. doi: 10.1371/journal.pcbi.1011164 (PMC10824453; doi:10.1371/journal.pcbi.1011164)
Supplement: S3 Table — The final model consisted of 78 of the NMMs as described above, which were coupled together based on the structural DTI network results from Gong et al. (2009). Coupling between two NMMs, if present, was always reciprocal, and excitatory. The output E(t) of the main excitatory neurons of one NMM was used as the input for the impulse response he(t) of the excitatory neurons of the second NMM; the output E(t) of the second module was coupled to the impulse response he(t) of the excitatory neurons of the first NMM. Coupling strength between neural masses was set at S = 1. A schematic illustration of the coupling between two NMMs is shown in S1 Fig. For the present study the model was extended in order to be able to deal with activity dependent degeneration of connection strength between multiple NMMs coupled according to human DTI connectivity. The effects of tDCS were introduced into the model by increasing (for cathodal stimulation) or decreasing (for anodal stimulation) Vd1 to 5 or 9 from the baseline value 7. This change in threshold potential has the opposite effect on the excitability of the pyramidal excitatory neuronal population of the affected neural mass. (DOCX) [file pcbi.1011164.s003.docx]

**S3 Table.** Overview of model parameters, from de Haan et al., 2012. [49] The final model consisted of 78 of the NMMs as described above, which were coupled together based on the structural DTI network results from Gong et al. (2009). Coupling between two NMMs, if present, was always reciprocal, and excitatory. The output E(t) of the main excitatory neurons of one NMM was used as the input for the impulse response he(t) of the excitatory neurons of the second NMM; the output E(t) of the second module was coupled to the impulse response he(t) of the excitatory neurons of the first NMM. Coupling strength between neural masses was set at S=1. A schematic illustration of the coupling between two NMMs is shown in S1 Figure. For the present study the model was extended in order to be able to deal with activity dependent degeneration of connection strength between multiple NMMs coupled according to human DTI connectivity. The effects of tDCS were introduced into the model by increasing (for cathodal stimulation) or decreasing (for anodal stimulation) Vd1 to 5 or 9 from the baseline value 7. This change in threshold potential has the opposite effect on the excitability of the pyramidal excitatory neuronal population of the affected neural mass.

| **Symbol** | **Interpretation** | **Value** |
| --- | --- | --- |
| *t* | Sample time | 0.002 s |
| *P(t)* | Subcortical input level to each neural mas | 550 spikes s^-1^ |
| *Noise* | Random fluctuations around average level of *P(t)* | 1.0 |
| *A h_e_(t)* | Amplitude of EPSP | 1.6 mV |
| *A h_i_(t)* | Amplitude of the IPSP | 32 mV |
| *a h_e_(t)* | Shape parameter of EPSP | 55 s^-1^ |
| *b h_e_(t)* | Shape parameter of EPSP | 605 s^-1^ |
| *a h_i_(t)* | Shape parameter of IPSP | 27.5 s-^1^ |
| *b h_i_(t)* | Shape parameter of IPSP | 55 s^-1^ |
| *g* | Parameter sigmoid function that relates membrane potential to impulse density | 25 s^-1^ |
| *q* | Parameter sigmoid function that relates membrane potential to impulse density | 0.34 mV^-1^ |
| *Vd1* | Threshold potential used in the sigmoid function that relates membrane potential to impulse density for main population of excitatory neurons | 7 mV |
| *Vd2* | Threshold potential used in the sigmoid function that relates membrane potential to impulse density for main population of inhibitory neurons | 7 mV |
| *C1* | Connection strength between main population of excitatory neurons and inhibitory neurons | 32 |
| *C2* | Connection strength between inhibitory neurons and main population of excitatory neurons | 3 |
| *S* | Gain factor for the coupling strength between different neural masses | 1 |
| *T* | Time delay factor for the coupling between different neural masses | 0.002 s |
