# Supplementary material for: Virtual neural network-guided optimization of non-invasive brain stimulation in Alzheimer’s disease
Source: PLoS Comput Biol. 2024 Jan 17;20(1):e1011164. doi: 10.1371/journal.pcbi.1011164 (PMC10824453; doi:10.1371/journal.pcbi.1011164)
Supplement: S1 Fig — A) Schematic presentation of single neural mass model. Abbreviations are described in S2 Table. The upper rectangle represents a mass of excitatory neurons, the lower rectangle a mass of inhibitory neurons. The state of each mass is modeled by an average membrane potential [Ve(t) and Vi(t)] and a pulse density [E(t) and I(t)]. Membrane potentials are converted to pulse densities by sigmoid functions S1[x] and S2[x]. Pulse densities are converted to membrane potentials by impulse responses he(t) and hi(t). C1 and C2 are coupling strengths between the two populations. P(t) and Ej(t) are pulse densities coming from thalamic sources or other cortical areas respectively. B) Coupling of two neural mass models. Two masses are coupled via excitatory connections. C) Essential functions of the model. The upper left panel shows the excitatory [he(t)] and inhibitory [hi(t)] impulse responses. The upper right shows the sigmoid function relating average membrane potential to spike density. (DOCX) [file pcbi.1011164.s004.docx]

**
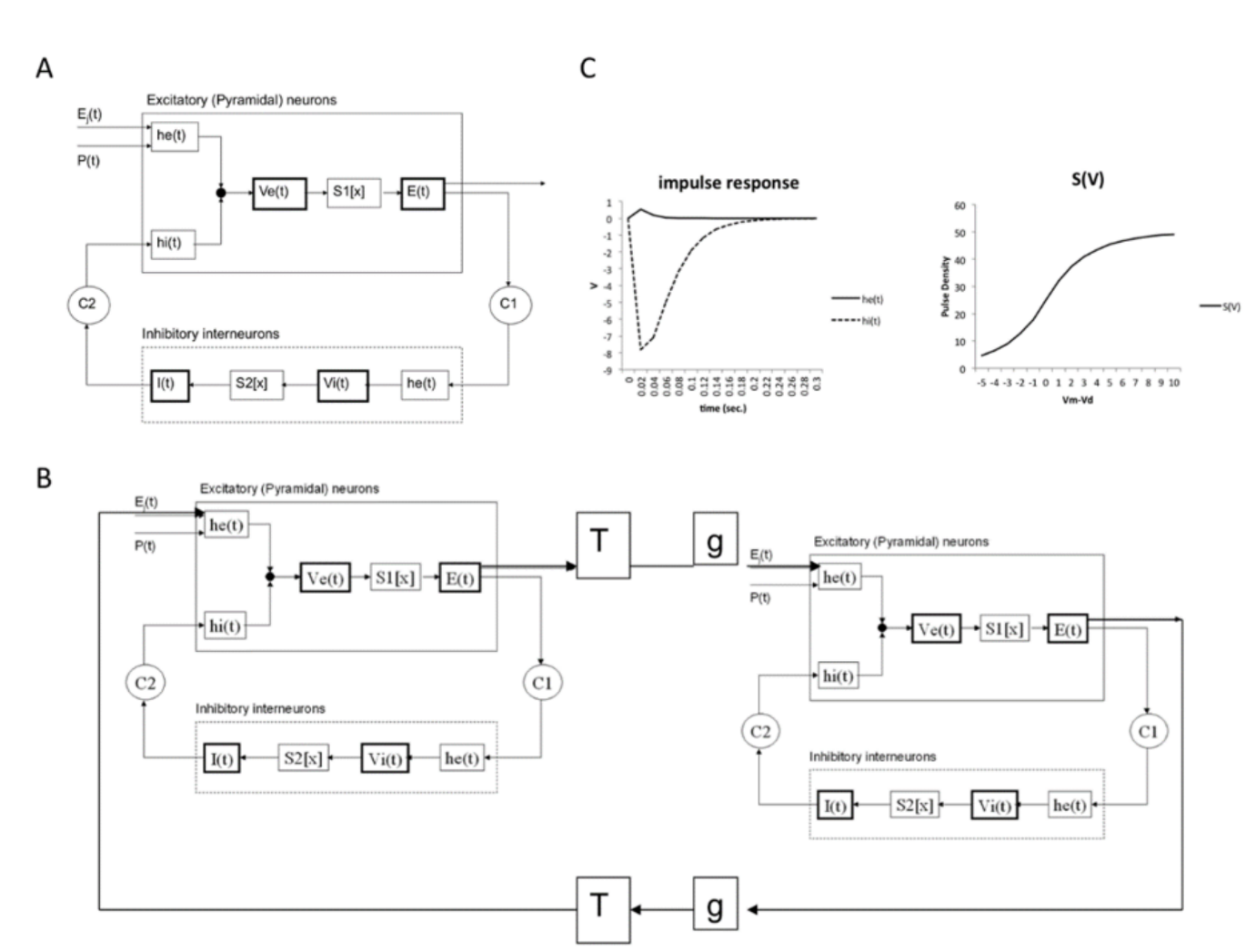
 S1 Fig.** Specifications of the neural mass model, from de Haan et al., 2012. **[49]**A) Schematic presentation of single neural mass model. Abbreviations are described in S2 Table. The upper rectangle represents a mass of excitatory neurons, the lower rectangle a mass of inhibitory neurons. The state of each mass is modeled by an average membrane potential [Ve(t) and Vi(t)] and a pulse density [E(t) and I(t)]. Membrane potentials are converted to pulse densities by sigmoid functions S1[x] and S2[x]. Pulse densities are converted to membrane potentials by impulse responses he(t) and hi(t). C1 and C2 are coupling strengths between the two populations. P(t) and Ej(t) are pulse densities coming from thalamic sources or other cortical areas respectively. B) Coupling of two neural mass models. Two masses are coupled via excitatory connections. C) Essential functions of the model. The upper left panel shows the excitatory [he(t)] and inhibitory [hi(t)] impulse responses. The upper right shows the sigmoid function relating average membrane potential to spike density.
