# Supplementary material for: Virtual neural network-guided optimization of non-invasive brain stimulation in Alzheimer’s disease
Source: PLoS Comput Biol. 2024 Jan 17;20(1):e1011164. doi: 10.1371/journal.pcbi.1011164 (PMC10824453; doi:10.1371/journal.pcbi.1011164)
Supplement: S2 Fig — A) Current flow modeling for the F7a-F4c setup at 2mA, with 5cm x 5cm electrodes using gel, carried out in the free SimNIBS software (Thielscher et al., 2015 [67]). The red square shows the anode position, while the blue square shows the cathode. B) Delineation (in red) of the triangular part of the left frontal inferior gyrus, corresponding to AAL region/neural mass 10. As more than 50% of AAL region 10 is contained in the electric field adjacent to the anode that exceeds 0.6 of total field strength (in this case 0.322), neural mass 10 in the model is considered anodally stimulated, and will have its excitability increased by lowering the threshold potential of the its excitatory pyramidal cell population Vd1 from default value 7 to 5. (DOCX) [file pcbi.1011164.s005.docx]

**
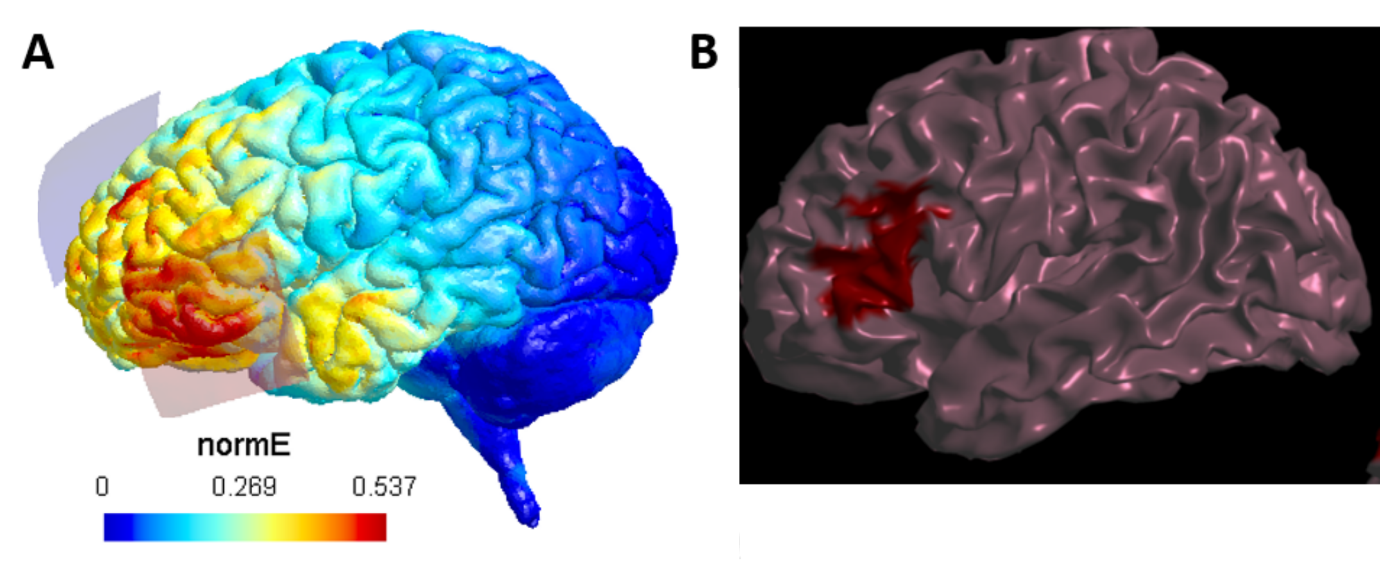
 S2 Figure:** Example of translating current flow modeling to AAL regions in the neural mass model. A) Current flow modeling for the F7a-F4c setup at 2mA, with 5cm x 5cm electrodes using gel, in SimNIBS software. The red square shows the anode position, while the blue square shows the cathode. B) Delineation (in red) of the triangular part of the left frontal inferior gyrus, corresponding to AAL region/neural mass 10. As more than 50% of AAL region 10 is contained in the electric field adjacent to the anode that exceeds 0.6 of total field strength (in this case 0.322), neural mass 10 in the model is considered anodally stimulated, and will have its excitability increased by lowering the threshold potential of the its excitatory pyramidal cell population Vd1 from default value 7 to 5.
