# Supplementary material for: Virtual neural network-guided optimization of non-invasive brain stimulation in Alzheimer’s disease
Source: PLoS Comput Biol. 2024 Jan 17;20(1):e1011164. doi: 10.1371/journal.pcbi.1011164 (PMC10824453; doi:10.1371/journal.pcbi.1011164)
Supplement: Attachment — Submitted filename: Response to reviewers.docx [file pcbi.1011164.s006.docx]

**Comments to the Authors:**

**Reviewer #1:** The authors utilized an established computational model of AD neurophysiology with an activity-dependent degeneration (ADD) algorithm. It models the AD-like brain network damage including early-phase neuronal hyperactivity and -connectivity and gradual oscillatory slowing and loss of connectivity. They used a systematic modeling approach to determine the optimal tDCS electrode positions in AD. They adjusted the threshold potential of the pyramidal neurons to simulate the tDCS effects on the neural mass. By comparing the performance outcome of 20 setups, they concluded that the contralateral parieto-frontal setups with anodal stimulation (PO7a-AF4c and PO8a-AF3c) resulted in the best performance. This study demonstrated how tDCS electrode montages would affect its effects on AD and their model can potentially guide and improve the tDCS therapy. Overall, the manuscript is well-written. However, there are some issues that need to be addressed.

**A: We thank the reviewer for the positive words and helpful comments. We hope to have addressed the questions sufficiently below.**

In Methods, it is not very clear how a specific neural mass is considered to be influenced by the tDCS. Pg 29: “All regions falling within the current flow area closer to the anode were considered to be excited, while to falling within the current flow area closer to the cathode were considered to be inhibited.” Also, in Discussion Pg 19, “we chose to include neural masses in the targeted population if there was current spread visible on approximately half or more of the associated AAL regions.” What does visible current spread mean? Do you have a particular cutoff or threshold for the amplitude of the electric field on the CFM at which you can say the neural mass was impacted? Did you also account for the variation in electric field strength between montages?

**A: AAL regions in which the electric field strength E was >0.6 of the maximum field strength were considered affected by the virtual tDCS, provided that this cutoff was exceeded in at least half of the AAL region. Two areas of influence could be delineated for all montages, associated with the anode and the cathode, determining whether the regions were considered to be excited or inhibited. All simulations were performed at a stimulation intensity of 2mA, and the maximum electric field was similar across montages, although there was large variation in how spread out the field was. In this study we chose for a binary system, where neural masses were considered to either be influenced or not. Ideally, it would indeed be most accurate to translate electric field strength to a change in excitability as a gradient. However, for this pilot study we chose to simplify this aspect, as this relationship is very challenging to quantify accurately, and would be even more challenging to relate to the excitability parameter in the model. Therefore, we kept the neural mass model parameter changes constant at a Vd1 change of +/-2, which was deemed large enough of a change to cause changes in the model, but not so large as to generate physiologically implausible activity. We acknowledge that this approach involves arbitrary choices, but we believe that the utilization of current flow modeling presents an improvement over simple anatomy-based assumptions.**

**Added to pg. 30:**

***“The electric fields were considered strong enough to influence associated AAL regions if the field strength exceeded 0.6 of the maximum field strength in at least 50% of the area of the region. The electric fields were assigned to either the anode or the cathode based on proximity, and AAL regions falling within the current flow area closer to the anode were considered to be excited, while to falling within the current flow area closer to the cathode were considered to be inhibited (See Supplementary Figure S5 for reference).”***

**Added the supplementary figure S5:**

***Supplementary Figure S5:*** *Example of translating current flow modeling to AAL regions in the neural mass model. A) Current flow modeling for the F7a-F4c setup at 2mA, with 5cm x 5cm electrodes using gel, in SimNIBS software. The red square shows the anode position, while the blue square shows the cathode. B) Delineation (in red) of the triangular part of the left frontal inferior gyrus, corresponding to AAL region/neural mass 10. As more than 50% of AAL region 10 is contained in the electric field adjacent to the anode that exceeds 0.6 of total field strength (in this case 0.322), neural mass 10 in the model is considered anodally stimulated, and will have its excitability increased by lowering the threshold potential of the its excitatory pyramidal cell population Vd1 from default value 7 to 5.*

**A**

**B**

Pg 10 and 11: In Figure 1EF and 2EF, the PLI and AEC curves before t = 10 are different between “AD without intervention” and “tDCS conditions”. Shouldn’t the results be the same for these conditions before stimulation onset?

**A: In our Lopes da Silva neural mass model there is still enough stochastic noise in the model (even after 100 iterations) to cause slight differences, despite the fact that all parameter settings are indeed the same before stimulation onset at t=10. For the technical motivation of this feature, we kindly refer the reviewer to the original publications (Lopes da Silva, 1974).**

There is an inconsistency in the naming of stimulation setups. When describing stimulation setups, you used “region_region” notation in tables (e.g., PO7a_AF4c) and “region-region” in figures (e.g., PO7a-AF4c). While in text, you mixed both notations.

**A: Fixed for consistency.**

Pg 27: In Table 2, include color bars for the electric fields on the head model when presenting the montages.

**A: Figures are now adapted to include color bar scales for electric fields.**

Discussion: Pg 16: “The functional connectivity patterns consistently featured an initial decrease in PLI and AEC at stimulation onset (Figures 1EF and 2EF).” This is not true. In Figure 1E, cathodal stimulation setups showed an initial increase in PLI.

**A: This sharp observation is correct, the text was adapted to account for this exception.**

**Page 16: “The functional connectivity patterns featured an initial decrease in PLI and AEC at stimulation onset, with the exception of PLI in the parieto-frontal setup (Figures 1EF and 2EF).”**

Discussion: Pg 17: “This could also help explain why the ipsilateral parieto-frontal setups (O1a-AF3c and AF3a_O1c) only resulted in modest improvement across outcome measures”. However, I did not find “ipsilateral parieto-frontal setups (O1a-AF3c and AF3a_O1c)” results in the manuscript. Is this a typo? Also, the notations for stimulation setups are in different formats.

**A: This was indeed a typo, and has been corrected. The correct montages are O1a-F3c and F3a-O1c. The results from this montage were only shown as their composite scores in Table 1, for the sake of brevity.**

**We would like to thank the reviewer again, and we appreciate the attention to detail, which certainly helped improve the clarity of the manuscript.**

**Reviewer #2:** In this study, Luppi et al employ a previously developed computational model of AD neurophysiology by their group to make predictions about optimal tDCS montages. The central aim was to develop a method for incorporating virtual tDCS interventions into their model and investigate the influence of different positions. While their attempt is very novel and laudable, the overall impact remains limited as the model itself has not been validated. So to make predictions of what electrode montage (placement) may work better than others when the underlying assumptions itself need to be corroborated, is on a loose footing.

**A: We thank the reviewer for the positive remarks and useful comments. We hope to have addressed them sufficiently below.**

1) Is it possible for the authors to somehow indirectly validate the model outputs ? By perhaps listing all tDCS studies conducted in AD, what outcome measures were studied, relating those outcome measures with the outcome measures of this study (relative psd, total spectral power, posterior alpha peak frequency, etc.). Because otherwise how would one trust the model output to be even relevant to the neurophysiology of the disease. For instance, why are other EEG bands (e.g. gamma) not relevant? This could be a new table.

**A: We thank the reviewer for raising this relevant point, and we acknowledge the importance of validating model output. In our opinion, model validation can be pursued in different ways. We would like to elaborate a bit on this here to provide the right context. For our study, we rely on neural mass models. These have been developed for several decades, and have shown to be able to produce realistic oscillatory patterns, where e.g. amplitude, phase and oscillation morphology and their interrelation have been extensively compared to human neurophysiological data. Note that the main strength of a neural mass model is not the exact absolute values of its output, but the natural relation between low-level parameters such as membrane potential, EPSP/IPSP shape and high-level output characteristics such as oscillatory firing rates. So the core of our model is an established description of plausible meso-scale neurophysiological dynamics. We then take this further by coupling multiple masses in a network, and introducing both network damage and therapy. A validation for the way damage and therapy are introduced is by choosing the model parameters (e.g. membrane potential to alter neuronal excitability) that most closely resemble the true mechanisms known from experimental literature. We have also published on the used damage algorithm (ADD) before, showing its capability to produce typical AD changes, and thereby providing some validation for this part (de Haan et al., 2012 and 2017).**

**An important validation method is indeed to compare model output to empirical data, and this will actually be the next phase in our project, for which we are already enrolling patients. However, it should be noted that here, the most important predictions the model makes concern the *direction* of change (e.g. slowing, decrease of functional connectivity), not the exact absolute values. Fitting model output values to empirical data is in our opinion not a good way of validation, as this is an overinterpretation of a neural mass model’s ability. As any brain dynamics model is necessarily a simplification of all underlying physiological processes, it is not expected to produce similar values in an absolute way. So, we primarily use the resulting *relative* changes in spectral power or functional connectivity as a measure of their success. In addition, we perform a visual check whether model output signals actually match true physiological EEG data (e.g. normal oscillatory waveforms, no flatlines or extreme amplitudes). This is an advantage of simpler models that do not describe biophysical oscillatory behavior, such as the Kuramoto or Stuart-Landau model.**

**Finally, because of the varying clinical tDCS study methodologies and results so far, comparing our model output to those studies seems less convincing to us than the model comparison to our upcoming model-guided human tDCS-MEG data. Again, while we do not expect reproduce absolute values, particularly because we would be comparing virtual time results to real time results, we aim to reproduce the direction of change in outcome measures in response to stimulation. Our main validation approach is described on page 21, we hope it adequately conveys our position:**

***“True validation of our model prediction requires replication in AD patients. This is planned to be done in the following clinical validation phase of this project, in which early-stage, bio-marker positive AD patients will undergo a simultaneous tDCS-MEG session, using our best performing stimulation setup. -- The goal of the clinical study will be to assess whether our model predictions match what is seen in real-time MEG of AD patients, and whether personalized approaches outperform a generalized one.”***

**We discuss the validity of the model itself on page 25:**

***“This neural mass model is an adjusted version based on the original by Lopes da Silva et al. [46], primarily developed to simulate a human alpha rhythm [59, 60]. It has been used extensively in related network studies on Alzheimer’s disease [47, 48, 61, 62]. A key feature of the model is that the excitatory and inhibitory populations making up the neural masses generate EEG/MEG-like output, relating neuronal circuit characteristics to cortical oscillatory activity.”***

**The role of the ADD algorithm in simulating AD is discussed on page 5:**

***“Moreover, the model features an activity-dependent degeneration (ADD) algorithm, which has been shown to resemble AD-like brain network damage, including early-phase neuronal hyperactivity and -connectivity and gradual oscillatory slowing and loss of connectivity [47]. By tuning the excitability of tDCS-targeted neural masses in the model, it can be used to systematically explore and make predictions of their effects on disrupted brain network activity [48, 49]. Using this setup, we can also examine whether treatment success is related to stimulating regions with specific network profiles, for example mainly stimulating hub regions, as suggested by existing literature [9].”***

The authors do mention that the model was primarily developed to simulate human alpha rhythm. If this is the case, the title and scope should be limited to AD outcomes related to alpha?

**A: The basic Lopes da Silva neural mass model mainly describes human alpha activity (not exclusively), and is capable of representing the entire 1/f human power spectrum. To us, this fits the focus on alpha power change (shift towards slower theta or higher alpha/beta) which is a relevant choice for our research question, because the most well-established oscillatory change in AD is a shift of relative alpha power (and the posterior peak frequency) to slower theta frequencies. Building on our previous answer, the absolute change in power is less important than the direction of change. As long as the model correctly predicts relative changes in these most relevant AD-sensitive markers, it can become useful in clinical practice. In our eyes, it is more important that a treatment in this AD damage model is able to restore oscillatory slowing, and less important exactly which quantitative power spectrum values it produces. Since we focus on relative change, and we can see and describe e.g. increases in relative theta power, we feel that limiting title and scope to alpha would not be entirely correct.**

2) Page 25- this “tDCS-MEG pilot study” - remove MEG.

**A: This has been removed, as it indeed described a later stage not yet included in this manuscript.**

3) Can the authors add more details on how the electrode placement parameter space was limited to 20 setups. Table 1 does show all the montages but not exactly clear how 20 is reached.
Do these 20 montages cover all literature of tDCS in AD?

**A: Text was adjusted to clarify that there are 4 asymmetrical and 2 symmetrical unique montages. Given that all unique montages were reversed in polarity and asymmetrical montages were additionally reversed hemispherically, there were in total 4x2x2 + 2x2 = 20 montages. This number of montages was chosen for a balance between the number of simulations that could be feasibly carried out, while covering relevant regions of interest. This limitation was necessary, as otherwise the total would amount to ±20^2^ options in the normal 10-20 EEG layout alone.**

**While we cannot claim to have covered all literature of tDCS in AD with these montages, we did aim to include the main targets for tDCS in AD such as the dlPFC and precuneus region, and also to include stimulation in all cortical lobes. The rationale for the chosen electrode placements are included in the rightmost column of Table 2.**

**Pg. 27: “*All unique electrode positions were varied to reflect either a change in polarity (switching the positions of the anode and cathode), contralateral hemisphere, or both. This resulted in four variations for each unique electrode position, except for the two bilaterally symmetrical montages, for which there were two variations each. These montages, with their electrode placements as well as the rationale behind each choice are described in Table 2.”***

**Added legend to Figure 2: *“The left column displays each unique electrode position used, and the middle column displays the associated current flow modeling done in SimNIBS [72]. The right column describes the intended target of interest, and the motivating literature.”***

4) while not critical, please include some additional information of the MRI dataset used for CFM- age, gender, etc.

**A: We used the freely available dataset labeled ‘ernie’, which is provided along with the SimNIBS software. This is based on a healthy young male, and has been used in other publications as an average brain. For the upcoming patient tDCS-MEG recordings we will also generate personalized strategies by embedding their individual structural anatomy & connectivity into the model.**

**Added more detail on pg. 30:“*We used a standard template of the young, healthy adult male brain provided with the software as the MRI input, along with specifications for 5x5 cm electrodes with impedance gel.*”**

5) fix typo in Fig 3B legend. Trace B is correlation between average composite score and DTI.

**A: The typo has been corrected.**

6) What does the weak significant correlation between average composite score and AEC and no correlation between average composite score and DTI imply?- especially in the context of model validity? Are there clinical studies of tDCS and AD in which functional connectivity was studied? And can the model be related to it, to demonstrate utility.

**A: The reviewer raises an interesting question. These correlations were post hoc analyses investigating possible explanations for why specific electrode montages were successful within the parameters of our study. This approach was motivated by recent literature finding promising results in non-invasive brain stimulation of the highly connected (“hub”) precuneus region in AD patients (Koch et al., 2022, Benussi et al., 2021 and 2022). In essence, we hypothesized post hoc that in our simulations, anodal stimulation of highly connected regions would lead to better performance in the form of the composite scores, as they can exert a stronger influence on the entire network. However, we did not find such a relationship in our dataset.**

**Pg. 17: *“While we hypothesized post hoc that strategies stimulating hub regions would be the most successful ones, we did not find strong correlations between connectivity of anodally stimulated regions and intervention success (Figure 3).”***

**If the reviewers question was actually more specifically about the differing relationships between AEC and DTI, we speculate that this may mean that stimulating a functional hub has different effects than stimulating a structural hub in this case. While the two types of connectivity are naturally related to each other, they are ultimately distinct measures functional and structural connectivity, respectively. As there are many reported structure-function dissimilarities throughout the literature, we would expect them here as well, and any difference would in our opinion not be a specific model validity concern.**

7) Please be consistent with labeling. In places “PO7” and “Po7” have been used.

**A: Text was double-checked to make sure the terminology is consistent.**

**We would like to thank the reviewer for the critical comments and insights. We hope to have satisfactorily addressed all concerns raised below, and to have made relevant improvements to the manuscript.**

**Reviewer #3:** This study presents a model of how TDCS mitigates changes in the brain network associated with AD. The authors integrate a virtual model informed by DTI connectivity with a current flow model to explore how varying electrode positions impact simulated values of relative band power, total power, and phase lag index. The study also investigates the correlation between the effect of TDCS and connectivity to assess whether hubs are the main drivers of this effect. While the computational study is intriguing, its translational value and the validation of the proposed approach remain unclear. My primary concerns are as follows:

**A: We thank the reviewer for the valuable comments and insights. We hope to have satisfactorily addressed the concerns raised below.**

1. The DTI connectivity data is derived from healthy controls. It's not made clear how the connectivity within an AD brain network is created. The assumption that AD connectivity can be simulated from controls may not hold true.

**A:** **We agree with the reviewer that this approach may have biased our findings. However, in this study we use a trusted AD network damage model to dynamically introduce structural and functional damage over time and before virtual stimulation onset (de Haan et al., 2012), which is an important factor when looking at stimulation effects over (virtual) time paths. The use of a static average AD template of structural connectivity would, in our view, be less realistic. However, we will introduced individual MEG-based connectivity of AD patients into the model when personalizing the simulations in the upcoming validation phase.**
2. The authors acknowledge that the results might be influenced by the size of ROIs. Is it possible for them to use the AAL atlas or any other atlas where brain regions are approximately of equal size, such as the AAL 300?

**A: For this study, we used the AAL atlas (Tzourio-Mazoyer et al., 2002). The non-uniform shape of the AAL regions is indeed one of our limitations. Confirmation of our results using another atlas with uniform region size would certainly be interesting. However, in the scope of this study, this would require redoing all simulations and analyses from scratch, which is not feasible. Furthermore, we do not expect that doing this would result in dramatically different neural mass inclusions, considering the widespread nature of the electric fields in two-electrode tDCS.**

3. Figures 1 and 2 are unclear as to what is meant by "shifting the values closer to healthy levels." The data visualization is convoluted - could they elaborate on the color map on the brain?

**A: We** agree with the reviewer that the clarity of these figures was suboptimal, and **have edited** **these figures and legends to improve their message. The definition of strategy success is explained in more detail in the methods section, which in our original manuscript came before the result section. A strategy was considered successful if it resulted in a shift of the outcome measure values towards healthy control values, in comparison to the AD condition without intervention. I.e. in Fig1C, the red lines denoting the anodal stimulation setups (denoted on the right), show an increase at stimulation onset, shifting the values closer to the healthy control values (green) in comparison to the no intervention condition (blue), therefore signifying the an improvement. The color maps on the brain show the electrode placement and electric field as determined by the current flow modeling, as a visual reference that is more intuitive than just the electrode locations.**

**To enhance the readability, we added to legends of Figures 1 and 2: “*Strategies were considered succesful if they resulted in a shift of the outcome measure values closer to the healthy control values (green) in comparison to the AD damage condition without intervention (orange). The brainplots show the electrode placement and current spread for all setups.*”**

4. Many of the connectivity-related changes could potentially be driven by subcortical structures. This study should recognize that subcortical structures are not taken into account and could be significant.

**A: This is a valid and interesting point, it is indeed true that the model used here does not include subcortical structures. We have added an account of this in the limitations. We do expect that the direct effects of tDCS are mainly limited to the cortex, but this is not the whole story. However, there is a parameter for subcortical (thalamic) input in the model, as we have now elaborated in our manuscript:.**

**Added to pg. 19: *“Another improvement would be to include subcortical structures of the AAL atlas used in the modeling. While the effects of tDCS mainly reach the cortex, this does not mean that there is no relevant interaction between the stimulated areas and subcortical regions. A more detailed incorporation of the subcortex (for example by using all subcortical ROIs of the full AAL atlas) in the model could improve its accuracy. Regarding model dynamics, the Lopes da Silva neural mass model does feature subcortical (thalamic) input as an important parameter to obtain realistic thalamo-cortical feedback loops for oscillatory behavior.”***

5. The authors should elucidate how they can validate their model simulation.

**A: Please refer two our answers for question 1 of reviewer #2 for a detailed response. In essence, we agree with the importance of model validation, and in this case it is relevant at the level of the model itself and at the level of our simulation results. The neural mass model itself has been published on previously and has been validated by demonstrating its capacity to reproduce neurophysiological changes in AD empirical data. The results of simulation will be validated using simultaneous tDCS-MEG sessions in AD patients, in which the tDCS montages are based on the best performing strategies in our simulations (see manuscript pg. 21).**

6. How were the other model parameters chosen? For reproducibility purposes, the exact selection of parameters should be clearly stated.

**A:** **All model parameters were either chosen as described in methods at page 31 and in Supplementary Table 2 or kept at default values in the software, which are also listed in the Supplementary Table 2. These default values are based on the original work by Lopes da Silva and others, to reproduce realistic oscillatory behavior. The same default values have been previously used to simulate oscillations, functional connectivity, AD damage and brain stimulation (see supplementary material from de Haan et al., 2012 and 2017).**

**We added another reference to the supplementary material about model parameters on pg. 32:**

***“Model settings are described in the Supplementary Materials S3 and S4, and were kept at default unless otherwise noted.”***

Minor point: Please correct the typo in the spelling of "threshold" in the method section.

**A: The typo has been corrected.**

**We sincerely thank the reviewer for the critical comments and insights. We hope to have satisfactorily addressed all concerns raised below, and to have made relevant improvements to the manuscript.**
