# Supplementary material for: Virtual neural network-guided optimization of non-invasive brain stimulation in Alzheimer’s disease
Source: PLoS Comput Biol. 2024 Jan 17;20(1):e1011164. doi: 10.1371/journal.pcbi.1011164 (PMC10824453; doi:10.1371/journal.pcbi.1011164)
Supplement: Attachment — Submitted filename: Response to reviewers 2.docx [file pcbi.1011164.s007.docx]

We sincerely thank the reviewer for the comments and hope to have addressed them sufficiently below.

**Reviewer #2: I am not sure whether authors responded to my question regarding relevance of other EEG bands (gamma). Why this was not an outcome measure of this study? Please add relevant text in paper.**

A: We agree with the reviewer that we could have stated more explicitly why we focus on specific frequency bands, and not on delta and gamma activity. We have chosen to focus on the dominant alpha rhythm due to its important role in AD progression. The model we use was originally fine-tuned for producing realistic oscillatory alpha range activity (Lopes da Silva 1974, Zetterberg 1978 and Stam 1999), which makes it well suited for our study goals. This does come with the trade-off that while more ‘distant’ frequency bands such as delta and gamma are less well represented. However, we do not believe this to be an issue, as gamma band EEG activity is not of the main resting-state EEG markers we use for describing the AD disease course in the clinic, and, like the delta band, it is much more prone to physiological (eye, muscle) artefacts.

To address this point more accurately, we have adapted our previous text:

Adapted on page 31: *“The frequency bands of interest were the theta (4-8 Hz), lower alpha (8-10 Hz), higher alpha (10-13 Hz) and beta (13-30 Hz) bands. As the model is mainly built around the alpha (8-13 Hz) band, it was the main frequency range of interest, while the adjacent frequency bands were investigated to assess the direction of any possible shift from alpha towards the theta or beta range. The delta and gamma bands are more artefact-prone and therefore less used as quantitative markers for AD diagnosis and effect monitoring in the clinic [78]. They are also less well represented in our neural mass model, which is why they were not included in our analysis.”*

We have extended a passage on page 23: *“This neural mass model is an adjusted version based on the original by Lopes da Silva et al. [46,47], which has been optimized for reproducing the human alpha rhythm [48, 61, 62]. While the model can be adjusted to reproduce the typical 1/f power spectrum, we chose to keep the model settings focused on the alpha band in order to study the decline of the dominant alpha rhythm in AD. The model has been used extensively in related network studies on Alzheimer’s disease, owing to the relevance of the alpha band in assessing oscillatory slowing in the disease [49, 50, 63, 64].”*

**It will be really helpful to the naive reader if the authors state in paper that the neural mass predictions help discern the direction of change and not the absolute values. In addition, authors should state their expectation for the future validation study- which is to predict direction of change.**

A: We agree that it is indeed helpful to stress this point more clearly in the paper, and have made the following additions to address this in the manuscript:

Added to page 6: *“We focused on assessing the direction of change, meaning that if AD-damage in the model causes oscillatory slowing in the form of reduced alpha power, a successful strategy would need to counteract this change and increase alpha power.”*

Extended a sentence on page 20: *“We aim to validate our model findings in an upcoming clinical tDCS-MEG intervention study, in which the model-predicted directions of change (and effect size) in outcome measures in response to tDCS will be compared those seen in empirical data.”*

Added a sentence on page 23: *“Instead of focusing on absolute values, which can be influenced by numerous choices in model settings and can lead to overfitting and over interpretation, we primarily used the model to assess directions of change in response to AD-damage and virtual tDCS, which it is well suited for.”*

From page 31: *“The frequency bands of interest were the theta (4-8 Hz), lower alpha (8-10 Hz), higher alpha (10-13 Hz) and beta (13-30 Hz) bands. As the model is mainly built around the alpha (8-13 Hz) band, it was the main frequency range of interest, while the adjacent frequency bands were investigated to assess the direction of any possible shift from alpha towards the theta or beta range. The delta and gamma bands are more artefact-prone and therefore less used as quantitative markers for AD diagnosis and effect monitoring in the clinic [78]. They are also less well represented in our neural mass model, which is why they were not included in our analysis.”*

**If the neural mass model is capable of representing the entire 1/f human power spectrum, please state so, explicitly in paper.**

A: We have extended a passage on page 23: *“This neural mass model is an adjusted version based on the original by Lopes da Silva et al. [46,47], which has been optimized for reproducing the human alpha rhythm [48, 61, 62]. While the model parameters, such the ones for postsynaptic potentials and threshold functions, can be adjusted to reproduce a more typical 1/f power spectrum, we chose to keep the model settings focused on the alpha band in order to study the decline of the dominant alpha rhythm in AD. The model has been used extensively in related network studies on Alzheimer’s disease, owing to the relevance of the alpha band in assessing oscillatory slowing in the disease [49, 50, 63, 64].”*

**Please explicitly mention that the Ernie dataset was used. The authors mention young. Please state the age range in paper.**

A: We have added the name of the dataset to the description. We did approach the software developers for detailed demographics, such as the age range, but they informed us that this data is not available due to complete anonymization of the dataset. However, while we agree that it is relevant to note that this is a template of the healthy young adult brain, we believe it to be more reliable as generic template: given the heterogeneity in atrophy patterns and co-pathology in old age and AD in particular, using an AD-patient-derived template would introduce more unwanted variability. However, please note that for our upcoming personalized strategies, we will customize the model using individual brain anatomy and connectivity.

Modified sentence on page 28: *“We used a standard template of the healthy young adult male brain (‘Ernie’) provided with the software as the MRI input, along with specifications for 5x5 cm electrodes with impedance gel.”*

The following references were added to the manuscript:

46.	Lopes da Silva FH, Hoeks A, Smits H, Zetterberg LH. Model of brain rhythmic activity. Kybernetik. 1974;15(1):27-37. doi: 10.1007/BF00270757.

48.	Stam CJ, Vliegen JHR, Nicolai J. Investigation of the dynamics underlying periodic complexes in the EEG. Biological Cybernetics. 1999;80(1):57-69. doi: 10.1007/s004220050504.

78.	Whitham EM, Pope KJ, Fitzgibbon SP, Lewis T, Clark CR, Loveless S, et al. Scalp electrical recording during paralysis: Quantitative evidence that EEG frequencies above 20Hz are contaminated by EMG. Clinical Neurophysiology. 2007;118(8):1877-88. doi: <https://doi.org/10.1016/j.clinph.2007.04.027>.
